# Perkin’s Metallic Tractors

**Published:** 1855-09

**Authors:** 


### PERKIN'S METALLIC TRACTORS.

Perkins and his Metallic Tractors excited at the close of the last century as much, if not more, attention than mesmerism and spiritualism have done in the latter half of the present; and found, we may add, advocates, and patrons, and believers, and dupes among the same classes:

> "Supported by a set of sturdymen,
> Dukes, quakers, doctors, lords and clergymen."

But Perkins was treated with far less consideration and civility by his professional associates than we find shown to the followers of Mesmer, Edmonds' spirits and the dealers in nostrums in the times that are.

The Medical Society of Connecticut, of which Dr. Perkins was a member, gave the following plain decision touching such abominations at their convention in Hartford, May 17, 1796:

"*Resolved*, Whereas Dr. Elisha Perkins, a member of this Society, having obtained a patent from under the authority of the United States, for the exclusive privilege of using and vending certain pointed metallic instruments, pretending that they were an invention of his own; and also that they possess inherent power of curing many diseases, which is contrary to the rules and regulations adopted by this Society, interdicting their members the the use of notrums: therefore *voted* that the *Elisha Perkins* be expelled."—*Amer. Med. Gaz.*

A righteous verdict.

